# Riverine tot-P loading and seawater concentrations in the Baltic Sea during the 1970s to 2000—transfer function modelling based on the total runoff

**DOI:** 10.1007/s10661-015-4538-y

**Published:** 2015-05-12

**Authors:** Jari Hänninen, Ilppo Vuorinen

**Affiliations:** Archipelago Research Institute, University of Turku, FI-20014 Turku, Finland

**Keywords:** Runoff, Phosphorus, Ecology of the Baltic Sea, Eutrophication, Transfer function modelling

## Abstract

The signal of climate through the North Atlantic Oscillation (NAO) extends to westerly weather and to the Baltic Sea river runoff (BSRR) and further to the salinity and the marine fauna in the Baltic Sea. Our working hypothesis was that increased BSRR should also lead to increasing nutrient concentrations in the seawater. In rivers, transfer function (TF) models of the loading were constructed by time series of BSRR and tot-P concentrations. Based on the loading time series, we modelled, to our knowledge, first time, seawater tot-P concentrations in both the Northern Baltic Proper and in the Gulf of Bothnia, both on the surface (0–20 m) and deeper (21–70 m) waters. Our results further suggest a unifying mechanism by the BSRR that could explain most prominent ecological changes observed in the Baltic Sea during and after the 1970s. Such changes are eutrophication (as in this paper) and decreasing salinity and growth and reproduction of marine fauna, all of which have been separately described as due to different causes. BSRR is crucial when possible future developments of the Baltic Sea environment are considered because a general opinion exists that the rainfall (and the BSRR) is expected to increase in pace with proceeding climate change.

## Introduction

We and others have earlier followed the signal of Atlantic climate in the pelagic ecosystem of the Baltic Sea. A series of modelling exercises was initiated by a study on the North Atlantic Oscillation (NAO) and the westerly weather related to the Baltic Sea river runoff (BSRR) and salinity (Hänninen et al. 2000). At the ecosystem level, we have studied zooplankton (Dippner et al. 2001; Hänninen et al. 2003; Vuorinen et al. 2004) and fish (Flinkman et al. 1998; Rajasilta et al. 2015). Regionally, we have modelled relations between BSRR and various climatic indices (Hänninen and Vuorinen 2010; 2012) and Baltic Sea plant and animal species distribution (Vuorinen et al. 2014). Our approach generally is to follow the effects of increasing runoff (during a selected period) of the 1970s, during which an increasing trend was shown by Hänninen and Vuorinen (2010) in ecosystem variables. Such variables include, among others, eutrophication (as in this paper) with increasing algal blooms, decreasing salinity and abundance of marine zooplankton and fish, e.g. cod, as well as decreasing size of the Baltic herring. All these phenomena have been separately described and analysed as being due to various environmental factors in, e.g. BACC (2008).

Questions posed by increasing eutrophication are crucial to be followed and eventually solved for the purposes of environmental protection, ecosystem planning and managing and the general welfare of an 80-million-people population around the Baltic Sea. Two large scale phenomena have been a central concept in the studies of the Baltic Sea nutrient concentrations. The central role of nutrient loading to the Baltic Sea has been assigned to BSRR in, e.g. HELCOM (2005; 2009) and modelled by e.g. Arheimer et al. (2012). A recently emerged special question in this case is the presence of “the largest desert in Europe”, which is a completely oxygen-free bottom water layer, besides including high amounts of highly poisonous hydrogen sulphide (Conley et al. 2009). The nutrients from this zone are constantly mixing into the bottom water layer (Viktorsson et al. 2013), occasionally with the incomes of the so-called Major Baltic Inflows (MBIs) (Eilola et al. 2014).

According to scenarios of the Intergovernmental Panel of Climatic Change (IPCC), Northern Europe will experience increased rainfall during the coming decades (Alcamo et al. 2007), which is likely to cause increased leaching of nutrients into the sea (Justić et al.^2003^; Graham 2004). This will further accelerate the Baltic Sea near shore eutrophication (BACC 2008; BACC II 2015). In this paper, our working hypothesis was that increased runoff (as foreseen by e.g., Meier et al. 2006; Neumann 2010; Philippart et al. 2011; a recent review by BACC II 2015) may even lead to increasing nutrient concentrations in the recipient water body. Our whole series of models would then demonstrate a unified mechanism to explain most of prominent ecological changes observed in the Baltic Sea during and after the 1970s.

## Materials and methods

### Study area

**Fig. 1:**
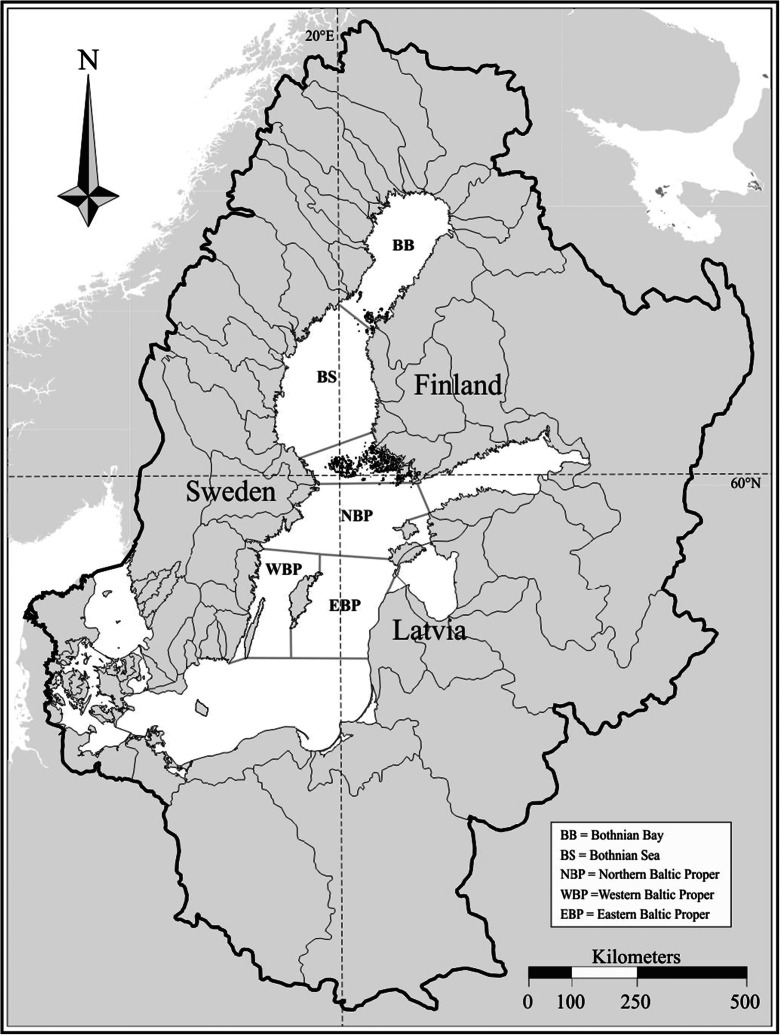
The total Baltic Sea with catchment area (*thick line*) and used subdivisions in modeling exercises. BB (Bothnian Bay) + BS (Bothnian Sea) = *the Gulf of Bothnia*, and NBP (Northern Baltic Proper) + WBP (Western Baltic Proper) + EBP (Eastern Baltic Proper) = *the Central Baltic Sea*

The semi-enclosed Baltic Sea is one of the largest brackish water basins of the world, with an area of 377,400 km^2^, but with a relatively small volume of 21,200 km^3^ (Fig. 1). The drainage basin size is 1,729,000 km^2^, which is about four times the area of the sea, itself. The mean water depth is only about 56 m (Voipio 1981). These special features of the Baltic Sea cause the quality of water to change according to possible changes in the watershed area. The origin of practically all incoming water to the Baltic Sea is from the Atlantic. Its salinity is maintained at an intermediary level by seawater intrusions from the North Sea through the Danish Straits. Saline water has a greater effect on the Southern Baltic and deeper water layers. Incoming freshwater originally evaporates in the Atlantic (in the constant high pressure area between Azores and Bermuda), enters the catchment area due to prevailing southwesterly winds, and after precipitation, finally reaches the Baltic Sea as freshwater runoff, which affects the surface water hydrography, especially in the northern parts of the Baltic Sea. The largest river in the region, the Neva, produces 18.2 % of the total runoff into the Baltic (Dietrich and Schott 1974; Grasshoff and Voipio 1981). For the period of 1950–1990, the mean annual river discharge into the Baltic Sea was 15,310 m^3^ s^–1^ (Hänninen and Vuorinen 2010; 2012). The measured record of total runoff into the Baltic Sea spans about 100 years, including a substantial rise during the 1920s and another one in the 1970s (Bergström and Carlsson 1994; Hansson et al. 2011). For a more detailed overview of the Baltic Sea oceanography, see Voipio (1981), Grasshoff and Voipio (1981) and Kullenberg (1981), respectively.

### Data

The study was conducted during the period from 1970 to 2000. Runoff data, provided by the Swedish Meteorological and Hydrological Institute (SMHI), were monthly values (km^3^) of total freshwater discharge from the catchment area into the Baltic Sea, divided into sub-drainage basins (Fig. 1), but excluding the Kattegat. The data comprised of both the monitored river runoffs and runoff estimates for non-monitored areas. Monitored runoff data represented about 200 river flow measuring stations covering 86 % of the area of the drainage basin. Runoff from the areas not monitored, mostly coastal areas between major rivers, was calculated using runoff from neighboring locations (according to Bergström and Carlsson (1994)). The total runoff for the Baltic Sea catchment was used to model tot-P, combining the areas of the northern, western and eastern Baltic Proper (hereafter, the central Baltic Sea), but the Bothnian Sea and Bothnian Bay drainage basins (hereafter, the Gulf of Bothnia) were modelled separately with runoff data for those basins only (Fig. 1).

Monthly loading (tons of tot-P) into the Baltic Sea were originally compiled in the 1970s and the 1980s by Stålnacke (1996), and completed in later years by several organizations and projects. The compiling system and data are documented by the project Marine Research on Eutrophication (MARE) in http://nest.su.se/bed/river_inputs.shtml. Nutrient concentration data also were loaded from MARE’s *Nest* (http://nest.su.se/nest).

Data were originally aggregated on a monthly basis, but in the Gulf of Bothnia, only during seasonal quarters due to less frequent monitoring. There were no missing observations in the runoff or tot-P loading, but in the hydrographical series, the number of missing observations varied between 5 and 25 %, depending on sampling area. However, averaging data from three adjacent sampling sites in the Baltic Proper and from two sites in the Gulf of Bothnia (Fig. 1), as well as into three depth zones, filled most of the gaps in the hydrographical time series. Hydrographical data sets were averaged over the sub-areas and pooled into three vertical water layers: from the surface to 21 m, 21 to 70 m, and below 70 m. This layering represented the vertical stratification, typical for the Baltic Sea. A permanent halocline, between 60 and 80 m, exists in the central Baltic Sea, and in summertime (June–September), and there also is a thermocline between 15 and 20 m (Kullenberg 1981). Due to the discharge of numerous rivers, a less saline surface water layer is also found above the thermocline. In the upper layer studied here, i.e. 0 to 21 m, runoff and tot-P loading were expected to have the greatest effect on the transfer function (TF) modelling of tot-P concentrations. The deeper layer, greater than 70 m, is more stagnant and has a constant higher salinity with low oxygen values and occasional hypoxia, especially in the middle areas of the Baltic Sea (Grasshoff and Voipio 1981). The deeper layer is mixed and aerated only by Major Baltic Inflows (MBIs), irregular intrusions of saline and oxygenated seawater through the Danish Straits, which, however, were largely absent during our study period (BACC 2008). We wanted to see if the development of the deepest water (>70 m) was different as compared to the shallower zones, as this would reveal possible importance of nutrients mixing from the sediment. The water layer between 21 and 70 m was considered a possible mixing zone, where the effects of both the discharge-enriched surface water and the deeper water could be present, simultaneously.

### Statistical analyses

Transfer function models, also called dynamic regressions, were applied using the Scientific Computing Associates (SCA) Statistical System Software, release 8.0 (Liu and Lattyak 2007). Transfer functions are able to connect one series not only with its own past values, but also with past and present values of other time series. The approach had already been tested and had proved useful in modelling relations between climate, hydrology, hydrography and biota of the Baltic Sea (Hänninen et al. 2000; Hänninen et al. 2003; Vuorinen et al. 2003; Vuorinen et al. 2004). We conducted analyses in two phases. First, we modelled the amount of tot-P loading into the Baltic Sea on the basis of total Baltic runoff. Then, we completed the analyses in detail by modelling tot-P concentrations in various depths and areas on the basis of tot-P loading time series.

All plausible models were compared and only one model was parsimonially chosen. The criteria for parsimony were as follows: The models we show have the smallest residual standard error among combinations of exploratory variables and are the simplest of obtained models (model with the lowest number of parameters), and show the highest proportional decrease in error term when the TF model residual standard errors were compared with those of the univariate ARIMA model of the same response variable (the decrease in error term was seen as due to inclusion of suitable exploratory information into the model).

## Results

**Fig. 2:**
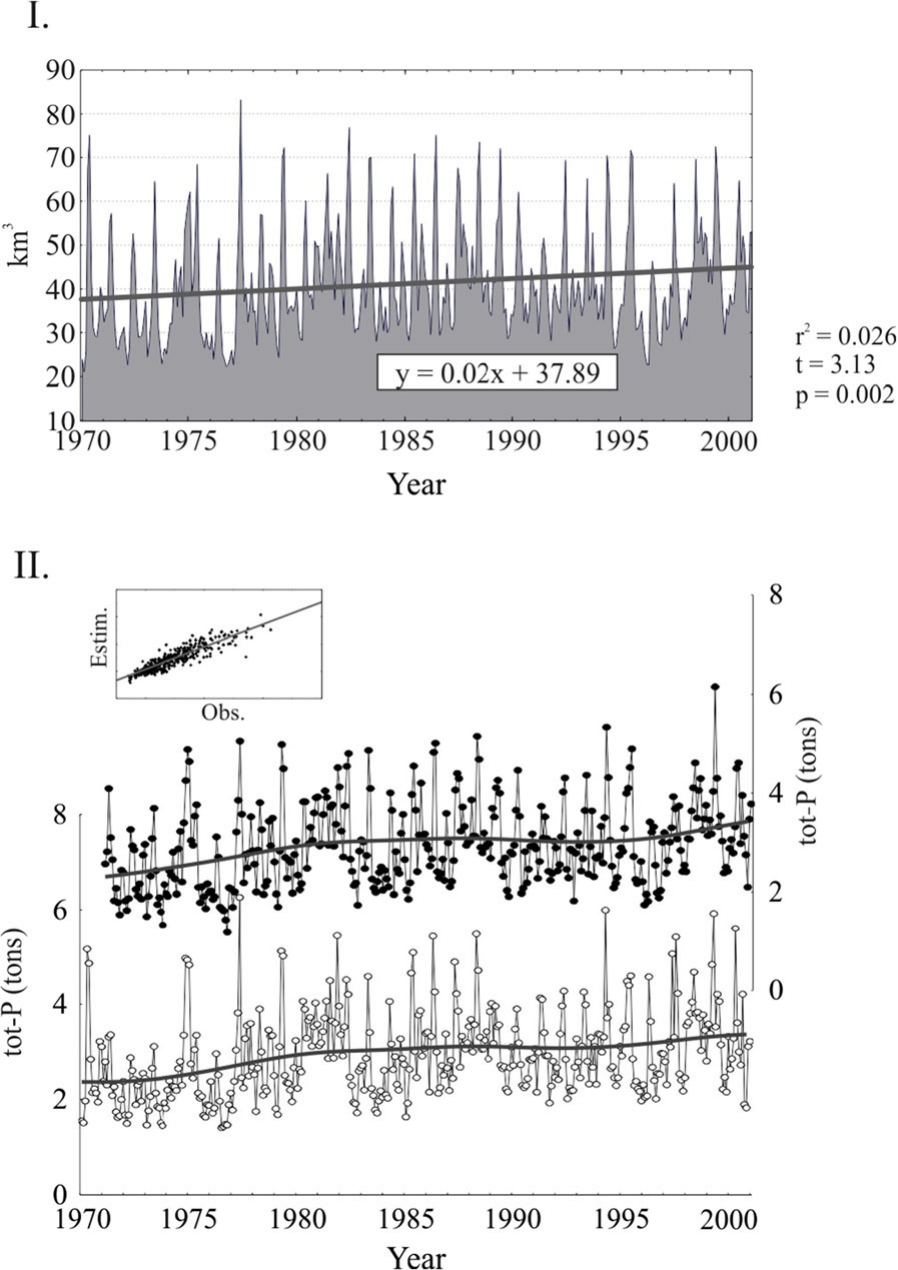
*I* Observed monthly runoffs, as deviations from overall monthly mean, into the Baltic Sea, during 1970–2000 according to Hänninen and Vuorinen (2010). The *thick line* in the observed series represents a linear function adjusted to the original runoffs for study period (*y* = 0.002x − 394.7, *r* = 0.1604; *F*(1320) = 9.77, *p* = 0.0019). The *thick line* in model panels indicates the estimated constant (overall monthly mean) runoffs. *II* Model for tot-P loading, on the basis of total freshwater runoff into the Baltic Sea. Modelled (*spots*) and observed changes (*open circles*) of the time series are based on the identified TF models. *Smooth lines* are drawn based on the distance-weighted least squares method. *Small boxes* in each panel are presented model fit scatterplots (observed values in the *X*-axis, estimated values in the *Y*-axis)

Arbitrarily selecting a period of increased runoff as the basis for our testing proved successful. This basically corroborated the idea of a close and regulative connection between BSRR and tot-P concentrations in various parts and depth layers of the Baltic Sea. Phosphorus transport into the Baltic Sea can generally be modelled with changes in the river runoff volume (Fig. 2, upper panel) and tot-P levels therein as the model revealed a close resemblance with measured and documented nutrient loading in the runoff (Table 1, Fig. 2, lower panel).

**Table 1:** Transfer function model for tot-P loading into the Baltic Sea

I. Total Baltic Sea tot-P loading vs. runoff	*r* ^2^ = *0*.84, *n* = 359 (1 − B^12^)tot ‐ Pt = *ω*_0_(1 − B^12^)Runoff_*t*_ + (1 − Θ_12_B^12^)/(1 − *Φ*_1_B_1_)*a*_*t*_		
Estimate	69.15	0.85	0.55
S.E.	2.77	0.03	0.05
*t* value	25.00	28.08	12.11
*p* value	<0.001	<0.001	<0.001

Initial estimates of the parameters with standard errors, *t* values and *p* values are shown. Coefficients of determination for the models are calculated with *r* ^2^ = 1 − [(*n* − 1)/(*n* − p)] [(sum of squares_resid._)/(sum of squares_total_)], where *n* = number of observations and *p* = number of estimated parameters. The time series data are monthly means. For more detailed description, see text

The freshwater runoff during the study period in the 1970s up to 2000 was clearly reflected in the TF model (Table 1, Fig. 2), with a lag shorter than 1 month (*ω*_0_ in Table 1), i.e. the resolution used in analysis (Fig. 2). It thus seems reasonable to expect increasing (tot-P) eutrophication to occur, should the expected increase of rainfall (and runoff) occur as a result of proceeding climate change. The trend which we expected on the basis of previously found trend in the runoff (Hänninen and Vuorinen 2010) was, however, not detected in the loading models.

**Fig. 3:**
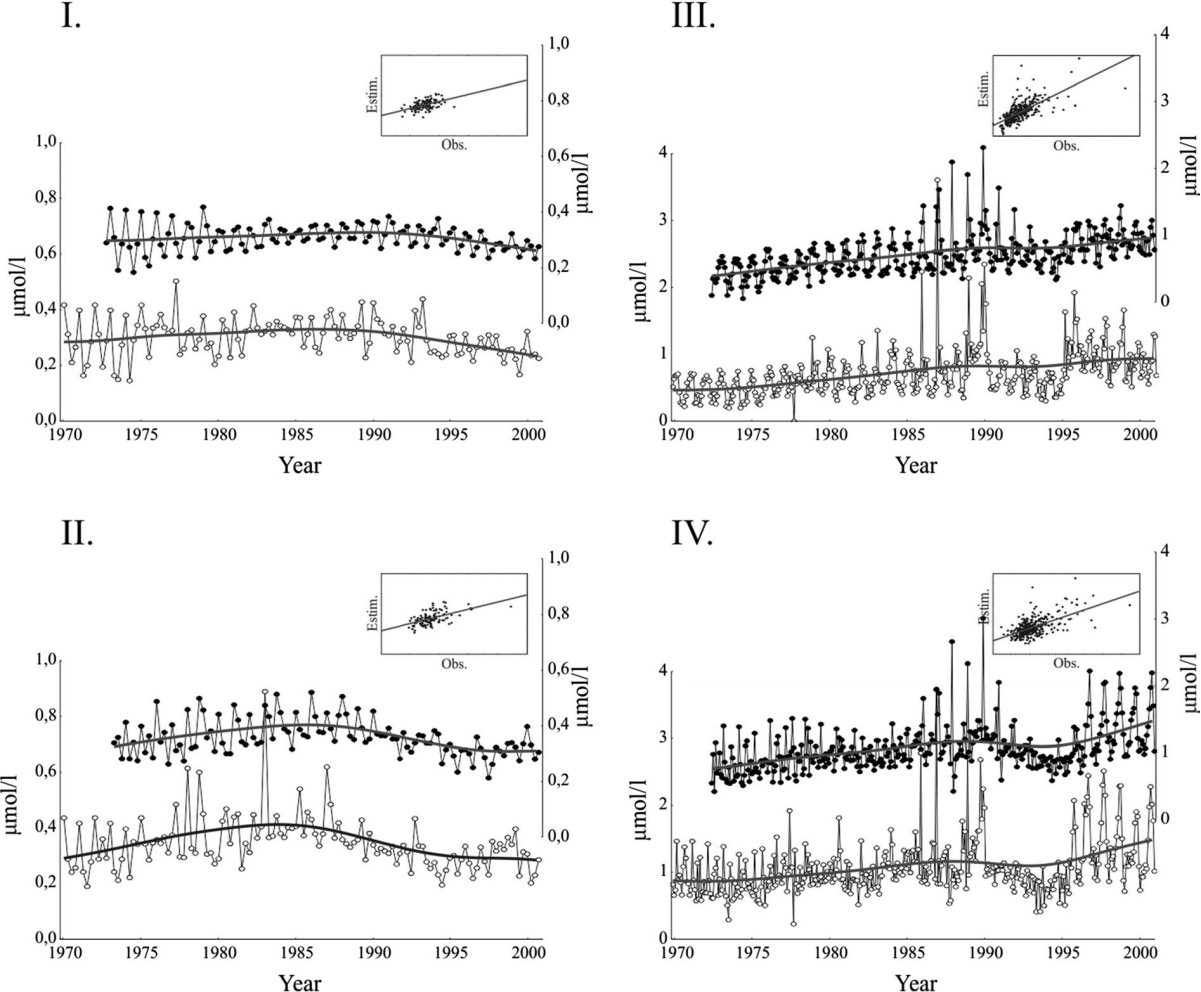
Models for tot-P concentrations in seawater on the basis of loadings. *I* Gulf of Bothnia, 0–21 m; *II* Gulf of Bothnia, 21–70 m; *III* Central Baltic Sea, 0–21 m; and *IV* Central Baltic Sea, 21–70 m; *Roman numerals* in the panels also refer to respective models in Table 2. On each panel, modelled (*spots*) and observed changes (*open circles*) of the time series are based on the identified TF models. *Smooth lines* are drawn with distance-weighted least squares method. *Small boxes* show model fit scatterplots (observed values in the *X*-axis, estimated values in the *Y*-axis)

We were able to model seawater concentrations of tot-P in the two uppermost water layers in both the Gulf of Bothnia and the central Baltic Sea on the basis of loading (Fig. 3, Table 2), and failed to do so only in the deeper (>70 m) layers. Somewhat contrary to our expectation also, the middle water layer (21 to 70 m) produced a plausible model. Thus, the tot-P concentrations for most of the water column in the Baltic Sea (the average depth is only 56 m) clearly follow the intensity and pace set by tot-P loading through the BSRR. Only tot-P concentrations the deepest basins that are below the constant halocline were impossible to model on the basis of tot-P loading through the BSRR, which suggests that they represent another regulatory mechanism that the concentrations closer to surface.

**Table 2:** TF models for tot-P concentrations in the seawater of the Gulf of Bothnia and in the central Baltic Sea

I. Gulf of Bothnia upper layer (0–21 m) tot-P loading vs. tot-P concentration	*r* ^2^ = 0.49, *n* = 113 $\left(1-{\mathrm{B}}^4\right)\mathrm{tot}\hbox{-} {\mathrm{P}}_{{\left(\mathrm{cone}.\right)}_t} = \left({\omega}_6{\mathrm{B}}^6\right)\left(1-{\mathrm{B}}^4\right)\mathrm{tot}\hbox{-} {\mathrm{P}}_{{\left(\mathrm{load}.\right)}_t}+\left(1-{\varTheta}_4{\mathrm{B}}^4\right){a}_t$			
Estimate	0.73	0.80		
S.E.	0.28	0.06		
*t* value	2.64	13.72		
*p* value	0.009	<0.001		
II. Gulf of Bothnia middle layer (21–70 m) tot-P loading vs. tot-P concentration	*r* ^2^ = 0.57, *n* = 111 $\left(1-{\mathrm{B}}^4\right)\mathrm{tot}\hbox{-} {\mathrm{P}}_{{\left(\mathrm{cone}.\right)}_t}=\left({\omega}_6{\mathrm{B}}^4\right)\left(1-{\mathrm{B}}^4\right)\mathrm{tot}\hbox{-} {\mathrm{P}}_{{\left(\mathrm{load}.\right)}_t}+\left(1-{\varTheta}_4{\mathrm{B}}^4\right)/\left(1-{\phi}_3{\mathrm{B}}^3\right){a}_t$			
Estimate	0.73	0.80	0.37	
S.E.	0.28	0.06	0.09	
*t* value	2.64	12.30	4.16	
*p* value	0.009	<0.001	<0.001	
III. central Baltic Sea upper layer (0–21 m) tot-P loading vs. tot-P concentration	*r* ^2^ = 0.82, *n* = 343 $\left(1-{\mathrm{B}}^{12}\right)\mathrm{tot}\hbox{-} {\mathrm{P}}_{{\left(\mathrm{cone}.\right)}_t}=\left({\omega}_{15}{\mathrm{B}}^{15}\right)\left(1-{\mathrm{B}}^{12}\right)\mathrm{tot}\hbox{-} {\mathrm{P}}_{{\left(\mathrm{load}.\right)}_t}\left(1-{\varTheta}_{12}{\mathrm{B}}^{12}\right)/\left(1-{\phi}_1{\mathrm{B}}^1\right)\left(1-{\phi}_2{\mathrm{B}}^2\right){\mathrm{a}}_t$			
Estimate	0.30	0.68	0.36	0.22
S.E.	0.13	0.04	0.06	0.06
*t* value	2.26	15.77	6.42	3.98
*p* value	0.024	<0.001	<0.001	<0.001
IV. Central Baltic Sea middle layer (21–70 m) tot-P loading vs. tot-P concentration	*r* ^2^ = 0.66, *n* = 343 $\left(1-{\mathrm{B}}^{12}\right)\mathrm{tot}\hbox{-} {\mathrm{P}}_{{\left(\mathrm{cone}.\right)}_t}=\left({\omega}_{16}{\mathrm{B}}^{16}\right)\left(1-{\mathrm{B}}^{12}\right)\mathrm{tot}\hbox{-} {\mathrm{P}}_{{\left(\mathrm{load}.\right)}_t}+\left(1-{\varTheta}_{12}{\mathrm{B}}^{12}\right)/\left(1-{\phi}_1{\mathrm{B}}^1\right)\left(1-{\phi}_2{\mathrm{B}}^2\right){a}_t$			
Estimate	0.40	0.60	0.23	0.21
S.E.	0.20	0.05	0.06	0.05
*t* value	2.40	13.04	4.21	3.84
*p* value	0.017	<0.001	<0.001	<0.001

Estimates of the parameters with standard errors, *t* values and *p* values. Coefficients of determination for the models are calculated with *r* ^2^ = 1 − [(*n* − 1)/(*n* − *p*)] [(sum of squares_resid._)/(sum of squares _total_)], where *n* = number of observations and *p* = number of estimated parameters. The series were monthly based in the central Baltic Sea series but quarterly in the Gulf of Bothnia. For more detailed description, see text

The time lags included in our models increased stepwise from nutrient loading models to runoff-concentration models and also differed between areas. The quarterly tot-P model in the Gulf of Bothnia included a time lag (*ω*_6_ in Table 2, panels I and II) of some 18 months between the loading and seawater concentrations in uppermost 0 to 21 m layer (noticing that in the quarterly series, one unit means 3 months). However, in the central Baltic Sea the effect of tot-P loading was observed in the uppermost seawater tot-P levels with a lag of about 15 to 16 months (*ω*_15 and 16_ in Table 2, panels III and IV), i.e. a slightly shorter lag than in the Gulf of Bothnia. Similar effects were found in the intermediate layers below the thermocline (21–70 m) in both of the areas, but not anymore in the deep water (>70 m) (Fig. 3, Table 2). The latter finding we interpret as effect of the P mixing from the bottom sediment which will cause that P concentrations in the water close to bottom do not reflect the same regulating factors (BSRR) as the surface water.

## Discussion

The relationship of runoff from the rivers, subsequent nutrient loadings, and their concentrations in the Baltic Sea remains unclear (Stålnacke 1996; Nausch et al. 1999; Voss et al. 2005a, b; Voss et al. 2011). Nitrogen and phosphorus enter the Baltic Sea as either waterborne or airborne inputs. During the past decade, waterborne inputs to the Baltic Sea constituted the main input of nitrogen (about 75 %) and phosphorus (about 97 %), while airborne inputs were only significant for nitrogen (HELCOM 2005). In 2000, these inputs amounted to 101 million tons of nitrogen and 34,500 tons of phosphorus (HELCOM 2005). The relation between runoff and loading is generally acknowledged (Fischer and Oppenheimer 1991; HELCOM 2005; HELCOM 2009). The sources of waterborne nitrogen were 58 % from agriculture and forestry, 32 % from natural backgrounds, and 10 % for point sources, while for phosphorus, the respective contribution percentages were 53, 27 and 20 % (HELCOM 2005). During the last 40–50 years, wintertime nitrogen inputs have increased fourfold and phosphorus levels eightfold in the Baltic Sea (Elmgren 2001; Larsson et al. 2001), while a decrease of inputs after that has also been reported (Gustafsson et al. 2012). For phosphorus, the BACC (2008) reports a significant increasing trend in the Baltic proper until 1983, followed by a plateau during the subsequent years. For nitrogen, an approximate doubling after a threshold around the 1970s occurred, but no statistical trend was reported (BACC 2008; Voss et al. 2011).

Our results suggest remote climate control of coastal and landlocked seas which have relatively large runoff areas. Different time lags between areas in this study corroborate our earlier observations, increasing lag resulting probably from longer snow cover period in the north (Hänninen and Vuorinen 2010). The existence of a lag might partially explain why so few studies have demonstrated a connection between runoff, loading and concentrations in the seawater. We suggest that there may be a further impact because human activities may affect changes in runoff itself by inducing global change. Our results also suggest that for the Baltic Sea, as a whole, the general increase of runoff, during the study period, may have masked regional and local effects of water protection measures (and land use) that would have had an effect of decreased loading and eutrophication. Our findings, taken together with earlier modelling and other studies (central publications in this respect are Hänninen et al. 2000 and Vuorinen et al. 2014) suggest that BSRR should be seen as one of the major environmental factors affecting the environmental status of the Baltic Sea. The length of observed time lags suggests further predictive modelling exercises when consequences of proceeding climate change are considered.

The observed runoff increase, since the 1970s, cannot be attributed to human induced climate change, but might instead be caused by internal variations of the climate system (Windsor et al. 2001; Hansson et al. 2011). Our results, even when they are based on a very short and selected period, generally support the relation between soil leaching and concentrations of total phosphorus in the sea. The overall increasing trend, which was demonstrated in the runoff, during the same period (Hänninen and Vuorinen 2010), was not reflected in the loading models, however.

In the Baltic Sea area, discussion of coastal environmental change has recently focused on two main topics: climate change and eutrophication, which are closely connected (Justić et al. 2003; Graham 2004; BACC 2008). Both regional and global climate models project increased winter rainfall in the north, and, in the Baltic Sea catchment area, the winter flows of rivers are expected to increase by up to 50 % (with opposite pattern in summer) (Voss et al. 2011; HELCOM 2013), subsequently coastal areas are expected to be eutrophied (e.g. Dippner and Ikauniece 2001). If an overall anthropogenic increase of precipitation and runoff can be expected, our results may, at first sight, suggest increasing eutrophication of the Baltic Sea. However, we think that predicting eutrophication effects in the distant future would be risky because of many complicating factors. First, in the hydrographical data from a 90-year analysis (from the 1920s to 1990s), the freshwater runoff to the Baltic shows large variations (Windsor et al. 2001). Secondly, because the actual concentrations of tot-P are generally lower in the north (see Fig. 3), the expected increase in winter precipitation in the northern part of the catchment might actually be counter-effecting the hypothesized eutrophication in the northern Baltic, due to a dilution effect by water from the Gulf of Bothnia running towards the Northern Baltic area. Furthermore, in a 500-year reconstruction of the Baltic Sea runoff, the total river runoff has decreased by 3 % per change in temperature increase (Hansson et al. 2011). A natural change in that effect would contradict our expectations of increased eutrophication due to anthropogenic reasons.

Considering the time period of 1980s to the present day, Stålnacke (1996) and Nausch et al. (1999) attributed changes in river loading to changes in land use. e.g. agriculture in the catchment area, and do not suggest much increase of loading over time. Stålnacke (1996) states that despite changes in land use, atmospheric deposition and wastewater treatment, the flow-normalized total river load of nitrogen and phosphorus in Sweden has been fairly constant since 1980. Nausch et al. (1999) reports that a reduction of 47 % in the phosphorus load from Denmark, since 1989, has resulted in significantly lower phosphorus concentrations, but no general changes in nitrogen have been seen. We did not flow-normalize our data, and our study suggests that important changes actually were possible before the 1980s as the increased runoff (Hänninen and Vuorinen 2010) may have increased the total nutrient loading into the sea. Looking into longer-term time series of rainfall over Sweden (Alexandersson 2004) and Northern Europe (Eisenreich 2005), it is evident that rainfall has been increasing over the last 100 to 150 years. The strong connection among runoff, loading and the tot-P concentrations in seawater, taken together with increased rainfall during the last 100 to 140 years (Alexandersson 2004; Eisenreich 2005), emphasize a possible long-lasting natural eutrophication of the Baltic Sea which, when added on top of the expected anthropogenic one, may imply that the predictions of future nutrient loading are too conservative. This would be the result in modelling, if the modellers assume a steady baseline with no natural increase in rainfall, which, however has been found in the Baltic Sea watershed.

Concluding, we show that riverine tot-P loading and seawater concentrations in the Baltic Sea, during the 1970s to 2000, are tightly coupled as demonstrated by TF modelling based on the total BSRR. As runoff is among the factors that are predicted (modelled) to increase together with proceeding climate change in the Baltic Sea area (e.g. BACC 2008; HELCOM 2013; BACC II 2015), our results (even taking into account the caveats in BACC II 2015) may suggest further eutrophication in the future Baltic Sea. As increased rainfall during the last 100 to 140 years (Alexandersson 2004; Eisenreich 2005), emphasizing a possible long-lasting natural eutrophication of the Baltic Sea which, when added to the expected anthropogenic one, may imply that the predictions of future nutrient loading are too conservative. The observation and understanding of the BRSS would give a possibility to foresee expectable changes in the Baltic Sea ecosystem (due to time lags in system). Our series of models demonstrate a large overall effect by the BSRR to the major ecosystem variables in the Baltic Sea. After the increase of the BSRR in our study period, such large changes have been demonstrated in eutrophication (algal blooms), salinity, marine fish growth and reproduction. So our study together with earlier studies suggests that BRSS should be treated as a major regulating ecological signal that affects the whole Baltic Sea ecosystem.
